# Long Noncoding RNA *HOXA11-AS* and Transcription Factor HOXB13 Modulate the Expression of Bone Metastasis-Related Genes in Prostate Cancer

**DOI:** 10.3390/genes12020182

**Published:** 2021-01-27

**Authors:** Aya Misawa, Yukihiro Kondo, Hiroyuki Takei, Toshihiro Takizawa

**Affiliations:** 1 Department of Molecular Medicine and Anatomy, Nippon Medical School, 1-1-5 Sendagi, Tokyo 113-8602, Japan; ayamsw@gmail.com; 2 Department of Urology, Nippon Medical School, 1-1-5 Sendagi, Tokyo 113-8602, Japan; kondoy@nms.ac.jp; 3 Department of Breast Surgical Oncology, Nippon Medical School, 1-1-5 Sendagi, Tokyo 113-8602, Japan; takei-hiroyuki@nms.ac.jp

**Keywords:** *HOXA11-AS*, HOXB13, long noncoding RNA, metastasis, prostate cancer

## Abstract

Long noncoding RNAs (lncRNAs) are emerging as critical regulators of gene expression, which play fundamental roles in cancer development. In this study, we found that *homeobox A11 antisense RNA (HOXA11-AS)*, a highly expressed lncRNA in cell lines derived from prostate cancer bone metastases, promoted the cell invasion and proliferation of PC3 prostate cancer cells. Transcription factor homeobox B13 (HOXB13) was identified as an upstream regulator of *HOXA11-AS.* *HOXA11-AS* regulated bone metastasis-associated C-C motif chemokine ligand 2 (CCL2)/C-C chemokine receptor type 2 (CCR2) signaling in both PC3 prostate cancer cells and SaOS2 osteoblastic cells. The HOXB13/*HOXA11-AS* axis also regulated integrin subunits (ITGAV and ITGB1) specific to prostate cancer bone metastasis. HOXB13, in combination with *HOXA11-AS*, directly regulated the *integrin-binding sialoprotein* (*IBSP*) promoter. Furthermore, conditioned medium containing *HOXA11-AS* secreted from PC3 cells could induce the expression of *CCL2* and *IBSP* in SaOS2 osteoblastic cells. These results suggest that prostate cancer *HOXA11-AS* and HOXB13 promote metastasis by regulation of CCL2/CCR2 cytokine and integrin signaling in autocrine and paracrine manners.

## 1. Introduction

The metastatic spread of tumor cells to vital organs is the primary cause of mortality in patients with cancer. Bone marrow is a common destination for many malignant cancers, including prostate cancer, breast cancer, thyroid cancer, lung cancer, bladder cancer, renal cell carcinoma, neuroblastoma, and melanoma [1]. Mammary malignant cells and osteoblasts can induce hydroxyapatite crystal (mineralized calcium phosphate) deposition within primary tumors, which suggests that they can generate a microenvironment that favors the crystallization of calcium and phosphate ions into bone-specific hydroxyapatite.

Homeobox (*HOX*) gene clusters constitute some of the most ancient and highly conserved multigene loci among eukaryotes [2]. HOX genes encode homeoproteins that are key components of master regulatory pathways and have been studied in detail over the past decade for their roles in the mechanisms underlying both organogenesis and oncogenesis [3,4]. Studies of *HOX* genes have primarily focused on protein-coding regions, but recent advances in genomics have revealed a variety of sense and antisense noncoding transcripts embedded within the *HOX* clusters and their flanking regions [5]. Long noncoding RNAs (lncRNAs) are transcripts longer than 200 nucleotides that demonstrate no protein-coding capacity [6]. They contribute to epigenetic activation and repression by opening large chromatin domains, maintaining the chromatin state, or modulating RNA interference-mediated silencing processes (e.g., by acting as molecular sponges through both cis and trans mechanisms) [7]. *Homeobox A11 antisense RNA* (*HOXA11-AS*) is a highly conserved lncRNA located in the HOXA gene cluster on chromosome 7p15.2, at the opposite strand of the protein-coding gene, *HOXA11*. *HOXA11-AS* initiates tumor formation and metastasis [8]; it functions as an oncogene in many types of cancers by regulating polycomb repressive complex 2 (PRC2) [9] and acting as a sponge for microRNAs [10,11].

In this work, we found that lncRNA *HOXA11-AS* was highly expressed in cell lines derived from prostate cancer bone metastases, where it promoted cell invasion and proliferation. Furthermore, we identified a homeobox B13 (HOXB13) transcription factor as an upstream regulator of *HOXA11-AS.* In cooperation with HOXB13, *HOXA11-AS* regulated the expression of chemokines, integrins, and related genes (e.g., *integrin-binding sialoprotein* (*IBSP*)) to promote the bone-specific metastasis of prostate cancer. We propose that prostate cancer cell-derived *HOXA11-AS* acts in a paracrine manner to modulate cytokine signaling in osteoblastic cells within the bone marrow milieu.

## 2. Materials and Methods

### 2.1. Cell Lines and Reagents

Cells were obtained from American Type Culture Collection (Manassas, VA, USA). PC3 and LNCaP cells were grown in RPMI medium. VCaP and DU145 cells were grown in Dulbecco’s Modified Eagle’s Medium with 4.5 g/L d-glucose. SaOS2 cells were grown in Dulbecco’s Modified Eagle’s Medium with 1.0 g/L d-glucose. All culture media were supplemented with 10% fetal bovine serum, and all cells were maintained at 37 °C in 10% O_2_ and 5% CO_2_. 

To generate PC3 cells overexpressing *HOXA11-AS* (A11AS-PC3 cells), *HOXA11-AS-204* (ENST00000522674.1) cDNA was synthesized using RNA purified from PC3 cells. cDNA was used as a template to amplify the *HOXA11-AS* sequence with primers listed in Table S1 The amplified sequence was cut with *HindIII* and *KpnI* restriction enzymes and inserted into the pcDNA3.1 vector (Thermo Fisher Scientific, Waltham, MA, USA). Then, PC3 cells were seeded into 6-well plates, transfected with *HOXA11-AS* vector or negative control pcDNA3.1 empty vector with Lipofectamine 3000 (Thermo Fisher Scientific), and treated with 500 mg/mL G418 (Sigma-Aldrich, St. Louis, MO, USA). Surviving cells were grown in G418-containing medium as stably transfected cells. 

### 2.2. RNA Interference

siRNAs were designed using siDirect version 2.0 (http://sidirect2.rnai.jp) and purchased from Sigma Genosys (Sigma-Aldrich). Mission siRNA Universal Negative Control #1 was used as negative control siRNA (Sigma-Aldrich). Cells were transfected with siRNAs using Lipofectamine RNAiMax transfection reagent (Thermo Fisher Scientific) at a final concentration of 20 nM, in accordance with the manufacturer’s protocol. The siRNA sequences are listed in Table S2. 

### 2.3. Invasion and Proliferation Assays

For invasion assays, cells were seeded in CIM-Plates (Agilent Technologies, Santa Clara, CA USA) at a concentration of 4 × 10^4^ cells/well. For proliferation assays, cells were seeded in E-Plates (Agilent Technologies) at a concentration of 6 × 10^3^ cells/well. siRNA transfection was performed in 6-well plates, 24 h before seeding. Cell growth was monitored for 72 h using the xCELLigence Real Time Cell Analysis System (Agilent Technologies). Cell invasion assays were carried out in triplicate.

### 2.4. Microarray Analysis

Microarray analysis was performed using human Clariom D Assays (Thermo Fisher Scientific). For data analysis, GO term and pathway analysis were performed using the Microarray Analysis Tool (Thermo Fisher Scientific). These data are available in the Gene Expression Omnibus database (GEO Accession No. GSE147710; https://www.ncbi.nlm.nih.gov/geo).

### 2.5. Plasmid Construction

Inserts for *HOXA11-AS* and *IBSP* luciferase vectors were amplified with the PC3 genome as template, using the primers listed in Table S1. The insert of *HOXA11-AS* luciferase vector containing the promoter region ranged from −1468 to +383. The insert of *HOXA11-AS* luciferase vector without the promoter region ranged from −367 to +383. Both inserts were cloned into pGL3-Basic luciferase vector (Promega, Madison, WI, USA) using *KpnI* and *HindIII* restriction enzyme sites. The promoter region of the *IBSP* luciferase vector ranged from −344 to +90. An insert of 269 bp within this region was cloned into the pGL3-Basic luciferase vector (Promega) with *KpnI* and *SacI* restriction enzyme sites, using primers listed in Table S1. 

### 2.6. qPCR Analysis 

The NucleoSpin RNA Plus Kit (Macherey-Nagel, Düren, Germany) was used for total RNA isolation. RNA concentration was measured with a NanoDrop Spectrophotometer (Thermo Fisher Scientific) or Quantus Fluorometer (Promega). First-strand cDNA was generated using the PrimeScript RT reagent kit III (Takara Bio, Shiga, Japan). Expression levels were quantified by qPCR using the KAPA SYBR FAST ABI Prism 2 × qPCR Master Mix (Applied Biosystems, Foster City, CA, USA) and 7300 and 7900 Real-Time PCR systems (Applied Biosystems). Relative mRNA levels were determined by normalization to *GAPDH* mRNA levels. Primers are listed in Table S3.

### 2.7. Luciferase Assays

Plasmids containing *HOXA11-AS* or *IBSP* promoters were constructed by inserting respective promoter regions into the pGL3-Basic luciferase vector (Promega), as mentioned above. Cells were seeded in 24-well plates at a density of 1 × 10^5^ cells/well. The following day, cells were co-transfected with 20 pmol siRNA and 300 ng luciferase reporter plasmid using Xfect microRNA Transfection Reagent (Takara Bio), in accordance with the manufacturer’s instructions. As a transfection efficiency control, pRL-Tk Renilla luciferase reporter plasmid (Promega) was co-transfected at 10 ng/plate. For luciferase assays, a Dual-Luciferase Reporter Assay System (Promega) was used in accordance with the manufacturer’s instructions. Luciferase activity was measured using GloMax luminometer (Promega) and normalized to the levels of Renilla luciferase activity.

### 2.8. ChIP Assay

ChIP assays were performed using the Simple ChIP Enzymatic Chromatin IP Kit (Agarose Beads), in accordance with the manufacturer’s protocol (Cell Signaling Technology, Danvers, MA, USA). PC3 cells seeded in 10 cm dishes were transfected with siRNA for 24 h. Cells were cross-linked with 1% formaldehyde for 10 min at room temperature and stopped by addition of 0.2 M glycine. Chromatin was digested with micrococcal nuclease to an average size of 150 bp; the reaction was stopped by addition of 0.5 M EDTA. Nuclear membranes were lysed by sonication with 8 sets of 10 s pulses, using a Diagenode Bioruptor (Diagenode SPA, Región de Valparaiso, Chile). After centrifugation had been performed, anti-HOXB13 (Cat# PA5-78327; Thermo Fisher Scientific), or Normal Rabbit IgG (Cat# 2729; Cell Signaling Technology) antibodies were added to lysates; the mixtures were rotated at 4 °C overnight. Protein beads were then added, and the lysates were rotated for 2 h. Beads were then washed, and DNA was eluted and treated with proteinase K (Cell Signaling Technology). DNA was purified with spin columns and used as template for qPCR. Fold enrichment relative to input levels was quantified by qPCR using the SYBR Green PCR master mix and 7300 Real-Time PCR system. The *RPL30* locus was used as the positive control. Primer sequences for ChIP qPCR are listed in Table S3.

### 2.9. Western Blotting

Whole-cell lysates were prepared using NP-40 lysis buffer. Protein concentrations were determined using the Bradford Assay (Thermo Fisher Scientific). Twenty milligrams of each protein lysate were loaded onto 4–20% precast polyacrylamide gels (Bio-Rad, Hercules, CA, USA), separated by electrophoresis, and electrotransferred to Sequi-Blot PVDF Membranes (Bio-Rad). Primary antibodies for Western blotting were anti-HOXB13 (Cat# PA5-78327; Thermo Fisher Scientific), anti-ITGAV (Cat# PA5-86575; Thermo Fisher Scientific), anti-ITGB5 (Cat# PA5-17260; Thermo Fisher Scientific), anti-ITGB1 (Cat# ab179471; Abcam, Cambridge, MA, USA) anti-ITGB3 (Cat# ab179473; Abcam), anti-ACTB (Cat# A5316; Sigma-Aldrich), anti-CCL2, clone 2D8 (Cat# MABN712; Merck Millipore, Burlington, MA, USA), and anti-IBSP (Cat# 5468; Cell Signaling Technology). Primary antibodies were detected by using appropriate horseradish peroxidase-conjugated secondary antibodies. Signals were then detected with an Enhanced Chemiluminescence System (GE Healthcare, Piscataway, NJ, USA) and blots were scanned using the Image Reader LAS-4000 (Fuji Film, Tokyo, Japan). Signal intensity was quantified using ImageJ software (RRID: SCR_003070; National Institutes of Health, Bethesda, MD, USA).

### 2.10. Gene Expression Omnibus Dataset Analysis

RNA sequencing data from VCaP and LNCaP cells lines, which were derived from prostate cancer bone and lymph node metastases, respectively (GEO Accession No. GSE82225), were analyzed to investigate lncRNA upregulation in prostate cancer bone metastasis. Gene expression was determined as the number of reads per kilobase of exon model per million mapped reads. Ingenuity Pathway Analysis (IPA) software was used for this investigation (QIAGEN Bioinformatics). 

A clinical sample dataset was downloaded from the NCBI Gene Expression Omnibus database (GEO Accession No. GSE74685). Custom Agilent 44K whole human genome expression oligonucleotide microarrays were used. The set included 149 castration-resistant prostate cancer tumors from 63 patients. Samples analyzed were bone (*n* = 20), lymph node (*n* = 69), lung (*n* = 22), liver (*n* = 21), and others (*n* = 17). Data were analyzed using the interactive web tool (GEO2R) and Excel software (Microsoft, Redmond, WA, USA).

The expression of cancer metastasis-associated lncRNAs (*MALAT-1*, *NEAT-1*, *HOTAIR*, and *HOXA11-AS*) in four prostate cancer cell lines (VCaP, PC3, LNCaP, and DU145) and a normal prostate epithelial cell line PNT2 was analyzed by comparison of exosomal RNA versus total RNA. A lncRNA array dataset from NCBI Gene Expression Omnibus (GSE81034) was used. Arraystar Human LncRNA Array v2.0 was scanned with Agilent DNA Microarray Scanner. Values represent raw signal intensities normalized in quantile method by GeneSpring GX v11.5.1 and adjusted by Combat program to remove batch effects.

### 2.11. Isolation of Extracellular Vesicles (EVs)

To assess whether *HOXA11-AS* was present in EVs, EVs were isolated from the cell culture supernatant of PC3 cells in accordance with the differential ultracentrifugation approach described by Thery et al. [12]. Briefly, PC3 cells (7 × 10^6^ cells) were cultured in exosome-free RPMI medium. After 48 h, cell culture supernatant (150 mL) was collected. Following the first 100,000 × *g* centrifugation, supernatant (i.e., “EV-subtracted supernatant”) was collected. After the second 100,000× *g* centrifugation, the resulting pellet was filtered through a 0.22 μm filter and then washed by resuspension in phosphate-buffered saline. EVs were subsequently purified by centrifugation at 100,000× *g* using a 30% sucrose cushion. The concentration and size distribution of the isolated EVs were analyzed using the NanoSight particle tracking system (LM10; NanoSight, Amesbury, Wiltshire, UK) [13]. qPCR was performed as described above.

### 2.12. Statistical Analysis

Statistical differences (*p* values) among groups were obtained using two-sided unpaired *t*-tests. All experiments were performed at least two or three times. *p* < 0.05 was considered statistically significant. Statistical analyses were performed using Excel (Microsoft).

## 3. Results

### 3.1. HOXA11-AS Is Highly Expressed in Cell Lines Derived from Bone Metastases

**Figure 1:**
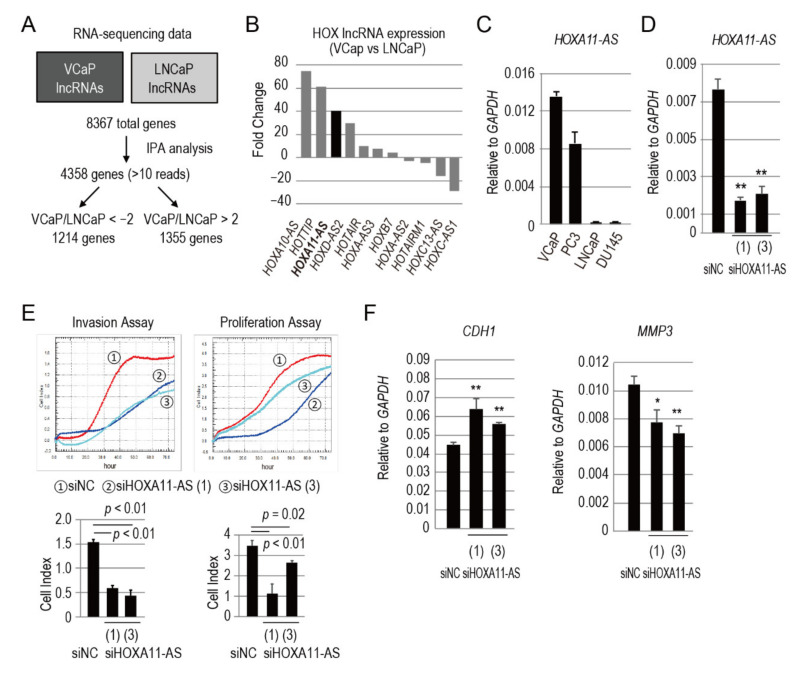
Identification of *HOXA11-AS* as a lncRNA highly expressed in bone metastasis-derived prostate cancer. (**A**) Flow diagram for the analysis of lncRNAs in VCaP and LNCaP cell lines. Sequencing data were downloaded from NCBI Gene Expression Omnibus (GSE82225). (**B**) Expression analysis of *HOX* cluster-derived lncRNAs in VCaP and LNCaP cell lines. Y-axis shows the fold change of the number of reads per kilobase of exon model per million mapped reads (RPKM) in VCaP cells with respect to the RPKM in LNCaP cells, calculated as the ratio between RPKM (VCaP)/RPKM(LNCaP) for each lncRNA shown in the X-axis. (**C**) *HOXA11-AS* expression analysis by qPCR in four different prostate cancer cell lines. (**D**) *HOXA11-AS* knockdown efficiency assessed by qPCR in PC3 cells transfected with siRNAs targeting *HOXA11-AS*. (**E**) xCELLigence real-time invasion assay of PC3 cells transfected with siRNA targeting *HOXA11-AS*; cell index represents invading cells at 48 h (left panel). xCELLigence real-time proliferation assay of PC3 cells transfected with siRNA targeting *HOXA11-AS*; cell index represents proliferating cells at 48 h (right panel). (**F**) *CDH1* and *MMP3* expression analysis by qPCR in PC3 cells transfected with siRNAs targeting *HOXA11-AS*. qPCR analyses were performed in triplicate (*n* = 3). Expression levels are presented relative to values of *GAPDH*, used as a reference gene. Values represent mean ± standard deviation. Statistical significance was determined by unpaired *t*-tests. *, *p* < 0.05. **, *p* < 0.01.

To investigate lncRNA upregulation in prostate cancer bone metastasis, we first analyzed existing RNA sequencing data from VCaP and LNCaP cells lines, which were derived from prostate cancer bone and lymph node metastases, respectively (GEO Accession No. GSE82225). Among the 8367 total genes, 4358 with more than 10 reads were selected from either VCaP or LNCaP cells by Ingenuity Pathway Analysis (IPA). The expression levels of these genes in both cell lines were compared; 1214 genes were expressed more than 2-fold and 1355 genes were expressed less than 2-fold in VCaP cells, compared with LNCaP cells (Figure 1A). Among the genes differentially expressed more than 2-fold in VCaP cells compared to LNCaP cells, we found out many lncRNAs derived from *HOX* clusters. We plotted these *HOX* cluster-derived lncRNAs in Figure 1B, where the X-axis shows the name of the lncRNA and the Y-axis shows the fold change of the number of reads per kilobase of exon model per million mapped reads (RPKM) in VCaP cells with respect to the RPKM in LNCaP cells, calculated as the ratio between RPKM (VCaP)/RPKM (LNCaP) (Figure 1B). *HOXA11-AS*, the 50th highly expressed gene among all lncRNAs identified in VCaP cells, was upregulated 40-fold, compared with LNCaP cells (Figure 1B). Expression of *HOXA11-AS* was verified in four prostate cancer cell lines (i.e., VCaP, PC3, LNCaP, and DU145) by quantitative polymerase chain reaction (qPCR) analysis (Figure 1C). The two cell lines derived from bone metastases, VCaP and PC3, showed substantially higher expression levels of *HOXA11-AS* compared with LNCaP and DU145, which are derived from lymph node and brain metastases, respectively. *HOXA11-AS* promotes cancer progression and metastasis in many types of cancers by the interaction with PRC2 or acting as a sponge for miRNAs [8,9,10,11]. Recently, another group already showed the function of *HOXA11-AS* in prostate cancer [14], but the underlying role of *HOXA11-AS* in prostate cancer metastasis still remains poorly understood. Therefore, we focused on this lncRNA by analyzing its upstream and downstream genes to determine genes involved in prostate cancer bone metastasis. We first tried to elucidate the relation between *AR* and *HOXA11-AS* using AR-positive VCaP cell line and found that *AR* knockdown by small interfering RNAs (siRNAs) did not show changes in *HOXA11-AS* expression (Figure S1). These data suggest that *HOXA11-AS* is not regulated by AR and lead us to proceed with AR-negative PC3 cell line for further experiments.

### 3.2. HOXA11-AS Promotes the Invasion and Proliferation of Prostate Cancer Cells

In most cancers, *HOXA11-AS* functions as an oncogene by promoting proliferation, migration, invasion, and metastasis. Therefore, in our first experiment, we compared invasive behaviors of human bone metastatic prostate PC3 cells between those transfected with *HOXA11-AS* specific siRNAs and those transfected with control siRNA, using the xCELLigence real-time analyzer system. Silencing efficiency was evaluated by qPCR analysis (Figure 1D). Under these experimental conditions, *HOXA11-AS* silencing led to a significant reduction in the invasive abilities of PC3 cells, compared with control cells (Figure 1E). Silencing of *HOXA11-AS* also led to a reduction in cell proliferation (Figure 1E). These findings suggest that *HOXA11-AS* promotes cell invasion and proliferation in prostate cancer cells. Furthermore, we analyzed the expression levels of metastasis-associated genes, such as *Cadherin-1* (*CDH1*, also known as *E-cadherin*) and *matrix metalloproteinase 3* (*MMP3*), in *HOXA11-AS*-repressed PC3 cells. *CDH1* expression increased, while *MMP3* expression decreased, in PC3 cells transfected with siHOXA11-AS for 48 h (Figure 1F). These data suggest that *HOXA11-AS* may regulate prostate cancer invasion through the modulation of metastasis-associated *CDH1* and *MMP3.*

### 3.3. HOXB13 Is an Upstream Regulator of HOXA11-AS

**Figure 2:**
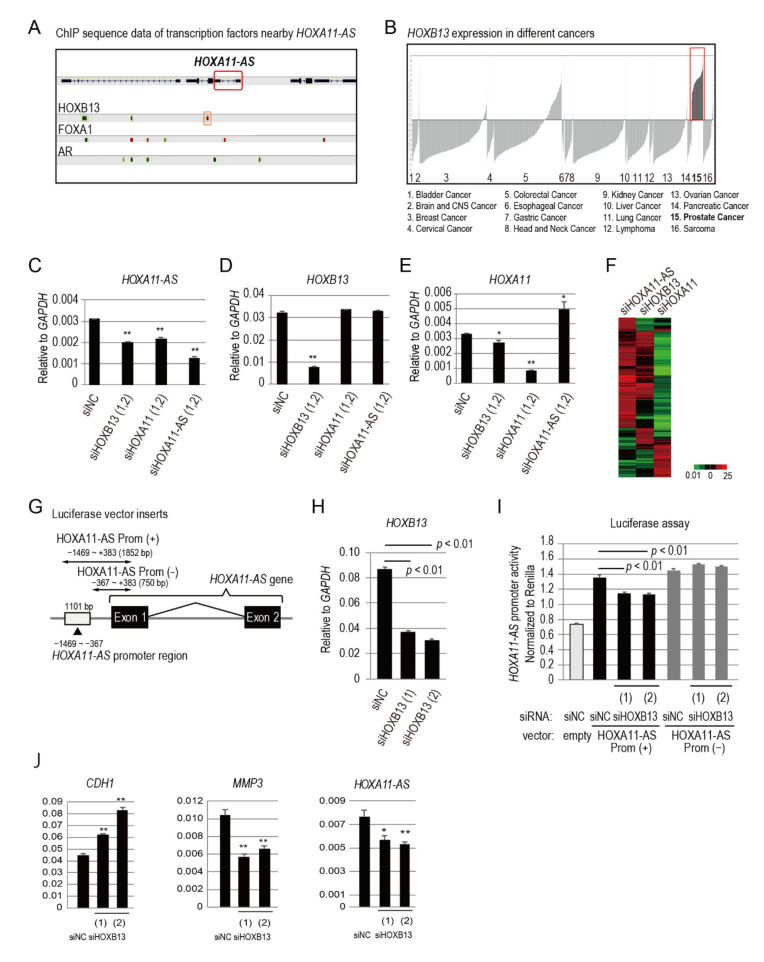
HOXB13 is an upstream regulator of *HOXA11-AS*. (**A**) Analysis of HOXB13 (orange box) and AR binding sites near *HOXA11-AS* gene (red box) using ChIP-Atlas database (https://chip-atlas.org/). The colors of peaks indicate the statistical significance values calculated by the peak-caller MACS2 (MACS2 scores). (**B**) Analysis of *HOXB13* gene expression levels in 16 different cancers using Oncomine database (https://www.oncomine.org/resource/login.html). *HOX13* expression levels in prostate cancer are indicated by a red box. Values on *y*-axis indicate log2 median-centered intensity, which ranges from 6.5 to −5.0. (**C**–**E**) Expression analysis by qPCR of PC3 cells transfected for 48 h with siRNAs targeting *HOXA11-AS*, *HOXB13*, and *HOXA11*. (**F**) Microarray analysis of PC3 cells transfected with siRNAs. Heatmap represents gene expression changes in PC3 cells transfected for 48 h with siRNAs targeting *HOXA11-AS*, *HOXB13*, and *HOXA11*. Red and green colors represent genes with increased (>2) and decreased (2<) expression levels, respectively, relative to negative control siRNA (siNC)-transfected cells. (**G**) *HOXA11-AS* exons, promoter region, and luciferase vector insert region are shown. HOXA11-AS Prom (+) includes the promoter region and part of exon 1 of the *HOXA11-AS* gene. HOXA11-AS Prom (−) covers the region after the promoter and part of exon 1. (**H**) Knockdown efficiency of PC3 cells transfected with siHOXB13. (**I**) Luciferase assay of PC3 cells co-transfected with siHOXB13 and luciferase vector, with or without HOXA11-AS promoter region (HOXA11-AS Prom (+) or HOXA11-AS Prom (−)). Vectors with siNC were used as control. Values were normalized to Renilla luciferase activity. (**J**) *CDH1*, *MMP3*, and *HOXA11-AS* expression analysis by qPCR in PC3 cells transfected with siRNAs targeting HOXB13. Data represent mean ± standard deviation. qPCR analyses were performed in triplicate (*n* = 3). Expression levels are presented relative to values of *GAPDH*, used as a reference gene. Values represent mean ± standard deviation. Statistical significance was determined by unpaired *t*-tests. *, *p* < 0.05. **, *p* < 0.01.

Next, we explored the upstream factors of *HOXA11-AS* in prostate cancer cells using the chromatin immunoprecipitation assay (ChIP)-Atlas database (https://chip-atlas.org/; accessed on 20 March 2019). In the genomic locus of *HOXA11-AS,* we found androgen receptor (AR)- and forkhead box A1 (FOXA1)-binding sites, which are key components in prostate cancer development and progression [15]. As mentioned above, we showed that *AR* knockdown did not affect the expression of *HOXA11-AS* expression in VCaP cells (Figure S1). We also examined whether *FOXA1* may regulate *HOXA11-AS* by siFOXA1, but *FOXA1* knockdown did not show changes in *HOXA11-AS* expression in VCaP cells (Figure S2). Near the *HOXA11-AS* gene locus, we also found a strong HOXB13-binding site; this homeoprotein is another important transcription factor and coregulator of AR [16] (Figure 2A). Furthermore, we analyzed *HOXB13* expression levels in different cancers using the Oncomine database (https://www.oncomine.org/resource/login.html; accessed on 28 February 2020). Among 16 different types of cancer, *HOXB13* was highly expressed in prostate cancer, suggesting its importance in prostate cancer progression (Figure 2B). Thus, we explored the relationship between *HOXB13* and *HOXA11-AS.*

First, we transfected PC3 cells with different siRNAs for 48 h and analyzed the expression levels of *HOXB13*, *HOXA11-AS*, and *HOXA11* by qPCR (Figure 2C,E,H). siRNAs targeting two different sequences were combined for more efficient gene silencing and downstream effects. *HOXA11-AS* was repressed by siHOXB13 transfection (Figure 2C). *HOXA11-AS* repression by siHOXB13 was also observed in VCaP cells (Figure S3). Transfection of siHOXB13 also reduced *HOXA11* expression (Figure 2E), suggesting that HOXB13 positively regulates both *HOXA11-AS* and *HOXA11* through a bidirectional promoter. Notably, siHOXA11-AS transfection enhanced *HOXA11* expression (Figure 2E). *HOXA11* has been identified as a novel tumor suppressor in renal cell carcinoma, which inhibits cell proliferation, migration, and invasion, while inducing apoptosis [17]. These results suggest that *HOXA11-AS,* as an oncogene, represses the opposite-strand tumor suppressor gene *HOXA11.* Unexpectedly, siHOXA11 transfection repressed *HOXA11-AS* expression, which suggests that *HOXA11* enhances *HOXA11-AS* (Figure 2C). A complex regulatory circuit among *HOXA11-AS*, *HOXA11*, and *HOXB13* may exist, and more experiments are needed in order to elucidate the exact mechanism of action among these genes. Neither siHOXA11 nor siHOXA11-AS transfection affected *HOXB13* expression (Figure 2D). Next, we performed microarray analysis of these siRNA-transfected PC3 cells using the Microarray Data Analysis Tool (version 3.2; Filgen, Aichi, Japan). Genes with expression levels that increased or decreased more than 2-fold, compared with their levels in negative control siRNA-transfected cells, were clustered and plotted (Figure 2F). As the heatmap shows, *HOXA11-AS* and *HOXB13* knockdown modulated similar genes, while *HOXA11* showed opposite results (Figure 2F). Common genes upregulated more that 2-fold in siHOXA11-AS and siHOXB13 samples compared to siNC were analyzed, identifying genes associated with cell membrane (Table S4). This suggests that *HOXB13* and *HOXA11-AS* share similar downstream signals related to cell membrane and cell to cell interaction. 

To examine whether HOXB13 is involved in *HOXA11-AS* regulation, we constructed a luciferase reporter vector containing the *HOXA11-AS* promoter, which includes a HOXB13-binding sequence; a vector excluding the promoter region was used as a control (Figure 2G). PC3 cells were co-transfected with siRNAs targeting *HOXB13* or negative control siRNA, as well as luciferase vectors with or without the *HOXA11-AS* promoter. *HOXB13* knockdown efficiency by siRNAs is shown in Figure 2H. siHOXB13 repressed *HOXA11-AS* promoter activity when transfected with the vector containing the HOXB13-binding region, while no change was observed upon transfection with the control luciferase vector lacking the *HOXA11-AS* promoter (Figure 2I). *CDH1* expression increased and *MMP3* expression decreased in PC3 cells transfected with siHOXB13, reinforcing HOXB13/HOXA11-AS axis (Figure 2J). Taken together, these results support the notion that *HOXB13* is an upstream regulator of *HOXA11-AS.*

### 3.4. HOXA11-AS Regulates C-C Motif Chemokine Ligand 2/C-C Chemokine Receptor Type 2 Signaling Associated with Prostate Cancer Bone Metastasis

**Figure 3:**
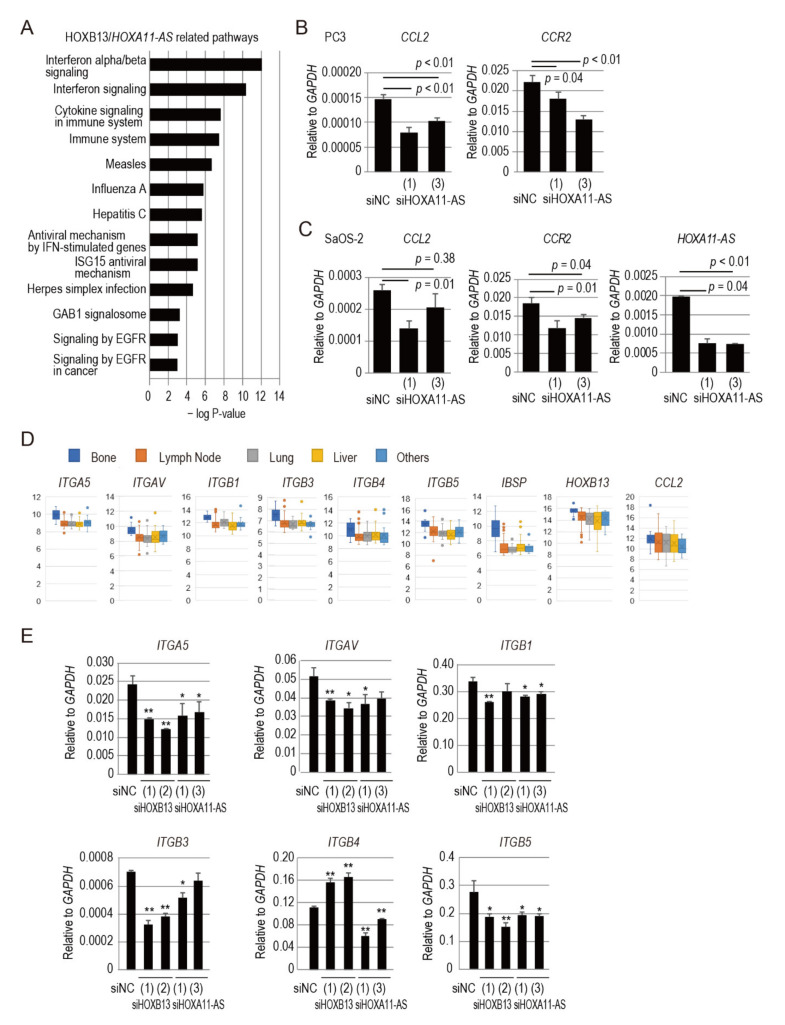
HOXB13/*HOXA11-AS* axis regulates CCL2/CCR2 signaling and integrin subunits specific to prostate cancer bone metastasis. (**A**) Pathway analysis of HOXB13/*HOXA11-AS*-regulated genes. Most relevant signaling pathways with lower *p* values are shown. (**B**) qPCR analysis of *CCL2* and *CCR2* in PC3 cells transfected for 48 h with siHOXA11-AS. (**C**) qPCR analysis of *CCL2*, *CCR2*, and *HOXA11-AS* in SaOS2 cells transfected for 24 h with siHOXA11-AS. (**D**) Expression analysis of different integrin subunits, *IBSP, HOXB13*, and *CCL2*, in castration-resistant prostate cancer clinical specimens that had metastasized to bone and other organs. A set of clinical samples from the NCBI Gene Expression Omnibus database (GEO Accession No. GSE74685) was analyzed. Values on *y*-axis represent batch-normalized log2 Cy3 signal intensity. (**E**) qPCR analysis of different integrin subunits (*ITGA5*, *ITGAV*, *ITGB1*, *ITGB3*, *ITGB4*, and *ITGB5*) of PC3 cells transfected for 48 h with siHOXB13 and siHOXA11-AS. qPCR analyses were performed in triplicate (*n* = 3). Expression levels are presented relative to values of *GAPDH*, used as a reference gene. Values represent mean ± standard deviation. Statistical significance was determined by unpaired *t*-tests. *, *p* < 0.05. **, *p* < 0.01.

We also performed Gene Ontology analysis of microarray data collected from PC3 cells transfected with siRNA targeting *HOXB13*, *HOXA11-AS*, or *HOXA11* using the Microarray Data Analysis Tool. The graph in Figure 3A shows the most relevant pathways, arranged according to their *p*-values; cytokine signaling and immune system pathways were the most affected pathways in *HOXB13* and *HOXA11-AS* knockdown prostate cancer PC3 cells. Previous studies have demonstrated the importance of different cytokines and their respective receptors in the progression of prostate cancer and bone metastasis (Table S5). In the bone marrow milieu, C-C motif chemokine ligand 2 (CCL2; also known as monocyte chemoattractant protein 1), the primary ligand for C-C motif chemokine receptor 2 (CCR2) [18], is produced in bone marrow osteoblasts, endothelial cells, stromal cells, and prostate cancer cells [19]. CCL2 is a prominent modulator of bone metastatic growth of prostate cancer [19], it promotes cell proliferation, invasion, and migration by binding to CCR2 in both autocrine and paracrine manners. Because the interaction between cancer cells and the host microenvironment is a vital component of tumorigenesis, the results of our microarray data analysis led us to focus on CCL2/CCR2 for examination of its relationship with *HOXA11-AS* in both prostate cancer cells and osteoblasts.

To investigate whether *HOXA11-AS* regulates *CCL2* and *CCR2*, we transfected siHOXA11-AS into PC3 cells and the human osteoblastic osteosarcoma cell line SaOS2. qPCR analysis showed that both genes were downregulated in PC3 and SaOS2 cells by *HOXA11-AS* knockdown (Figure 3B,C). We also analyzed changes of *CCL2* and *CCR2* expression after siHOXB13 silencing in PC3 and SaOS2 cells. *HOXB13* knockdown repressed *CCR2* in both cell lines, while *CCL2* was unchanged in these conditions, suggesting that *HOXB13* enhances at least *CCR2* expression (Figure S4). *CCL2* expression may depend not only on *HOXB13*, and further analysis are needed to elucidate what other factors may be involved in the regulation of this gene. Taken together, these data suggest that *HOXA11-AS* is an upstream regulator of CCL2/CCR2 signaling in both prostate cancer cells and osteoblastic cells.

### 3.5. HOXB13/HOXA11-AS Axis Regulates Integrin Subunits Specific to Prostate Cancer Bone Metastasis

Chemokines activate integrins by rapidly triggering inside-out signaling that regulates the binding of intracellular effector proteins to integrin cytoplasmic domains [20]. First, we analyzed a set of clinical samples from the NCBI Gene Expression Omnibus database (GEO Accession No. GSE74685), which includes 149 castration-resistant prostate cancer tumors from 63 patients. We compared the expression levels of eight integrin subunits (*ITGA2*, *ITGA5*, *ITGAV*, *ITGB1*, *ITGB3*, *ITGB4*, *ITGB5*, and *ITGB6*), *HOXB13*, *HOXA11-AS*, *CCL2*, *CXCL12*, and *IBSP* in prostate cancer tissues that had metastasized to bone, lymph nodes, lungs, liver, and other organs. Among the eight integrin subunits analyzed, *ITGA5*, *ITGAV*, *ITGB1*, *ITGB3*, *ITGB4*, and *ITGB5* were upregulated in the bone compared with the other organs, suggesting that some integrins may confer osteotropic properties on metastatic prostate cancer (Figure 3D).

To verify whether *HOXB13* and *HOXA11-AS* regulate integrin expression in prostate cancer, the expression levels of the six integrin subunits were analyzed in PC3 cells transfected with siRNA targeting *HOXB13* or *HOXA11-AS* by qPCR analysis. siHOXB13 transfection significantly downregulated most of the integrin subunits analyzed, with the exclusion of integrin *ITGB4*, which was significantly increased upon *HOXB13* knockdown (Figure 3E). siHOXA11-AS transfection also significantly downregulated these integrin subunits (Figure 3E). Next, the protein expression levels of the six integrin subunits were analyzed by Western blotting analysis. *HOXB13* silencing repressed ITGAV and ITGB1, whereas it induced ITGB4 protein expression (Figure S5). These results were substantially correlated with the findings in the PCR experiments (Figure 3E). Repression and partial repression of ITGAV and ITGB1 proteins, respectively, were also observed with siHOXA11-AS transfection (Figure S5). Furthermore, ITGB5 induction by siHOXA11-AS was observed. These results suggest that the HOXB13/*HOXA11-AS* axis modulates the expression of specific integrin subunits (i.e., ITGAV and ITGB1) in prostate cancer PC3 cells.

### 3.6. IBSP Promoter Is Directly Regulated by HOXB13 in Combination with HOXA11-AS

**Figure 4:**
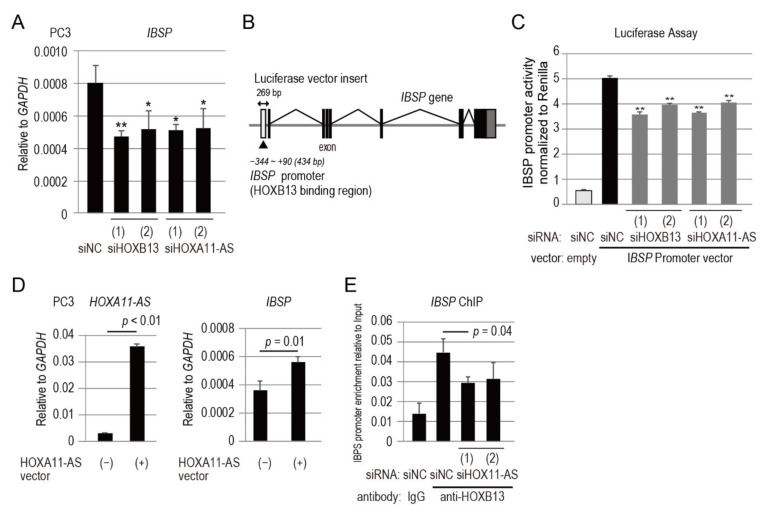
*IBSP* promoter is directly regulated by HOXB13 in combination with *HOXA11-AS*. (**A**) *IBSP* expression analysis by qPCR in PC3 cells transfected for 48 h with siHOXB13 or siHOXA11-AS. (**B**) *IBSP* exons, promoter region with HOXB13 binding site, and luciferase vector insert are shown. (**C**) Luciferase assay of PC3 cells co-transfected with siHOXB13 or siHOXA11-AS and luciferase vector. Vectors with negative control siRNA (siNC) were used as controls. Values were normalized to Renilla luciferase activity. Data represent mean ± standard deviation. (**D**) Expression analysis of *HOXA11-AS* and *IBSP* by qPCR in PC3 cells transfected for 48 h with or without *HOXA11-AS* expression vector. (**E**) ChIP analysis of HOXB13 in *IBSP* promoter region in PC3 cells transfected for 24 h with siHOXA11-AS. IBSP promoter enrichment relative to input was measured by qPCR. qPCR analyses were performed in triplicate (*n* = 3). Expression levels are presented relative to values of *GAPDH*, used as a reference gene. Values represent mean ± standard deviation. Statistical significance was determined by unpaired *t*-tests. *, *p* < 0.05. **, *p* < 0.01.

Notably, *IBSP* was significantly highly expressed in bone metastases, compared with metastases to other organs, in the dataset of clinical samples (Figure 3D). IBSP is a highly sulfated, phosphorylated, and glycosylated protein that constitutes a major component of the mineralized bone matrix [21]. Bone-derived *IBSP* plays important roles in breast cancer metastasis through induction of tumor cell seeding into the bone [22]. However, the mechanism by which *IBSP* is regulated in prostate cancer cells has not yet been elucidated. Thus, we performed qPCR analysis of *IBSP* expression in PC3 cells transfected with siRNA targeting *HOXB13* or *HOXA11-AS* to investigate whether HOXB13 and *HOXA11-AS* regulate *IBSP* expression in prostate cancer. Both siRNAs significantly downregulated *IBSP* expression in PC3 cells (Figure 4A).

The Ensembl database showed that the *IBSP* promoter harbors a HOXB13-binding site in its regulatory region (Figure 4B). To verify that HOXB13 directly regulates *IBSP* through its promoter, we constructed a luciferase vector containing the putative HOXB13-binding site (Figure 4B). A luciferase vector containing the *IBSP* promoter and siRNAs targeting *HOXB13* or *HOXA11-AS* were co-transfected into PC3 cells. As shown in Figure 4C, siHOXB13 knockdown significantly repressed *IBSP* promoter activity. These results demonstrated that HOXB13 directly regulated *IBSP* expression through its promoter. Furthermore, siHOXA11-AS knockdown also significantly repressed *IBSP* promoter activity (Figure 4C). *HOXA11-AS* overexpression enhanced *IBSP* expression in PC3 cells (Figure 4D). To determine whether *HOXA11-AS* was involved in regulation of the *IBSP* promoter by HOXB13, a ChIP assay using an anti-HOXB13 antibody was performed with PC3 cells transfected with siHOXA11-AS. HOXB13 binding to the *IBSP* promoter was markedly reduced in *HOXA11-AS* repressed cells (Figure 4E). Taken together with the findings that siHOXA11-AS knockdown did not affect the expression of *HOXB13* in PC3 cells (Figure 2D), these results suggest that *HOXA11-AS* upregulates *IBSP* expression through its promoter as a HOXB13-associated lncRNA.

### 3.7. HOXA11-AS Secreted from Prostate Cancer Cells Modulates the Expression of CCL2 and IBSP in Osteoblastic Cells

**Figure 5:**
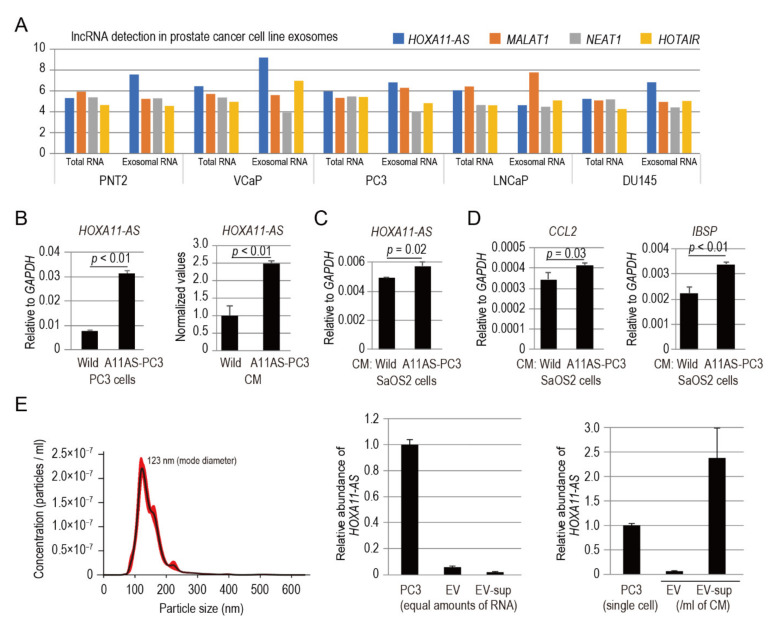
Prostate cancer PC3 cell-derived *HOXA11-AS* modulates *IBSP* and *CCL2* expression in SaOS2 osteoblastic cells in a paracrine manner. (**A**) The expression levels of well-known, cancer metastasis-associated lncRNAs (*MALAT-1*, *NEAT-1*, *HOTAIR*, and *HOXA11-AS*) in exosomes of prostate cancer cell lines by comparison of exosomal RNA versus total RNA. A lncRNA array dataset from NCBI Gene Expression Omnibus (GSE81034) was used; values represent raw signal intensities. (**B**) *HOXA11-AS* expression of PC3 stable cells overexpressing *HOXA11-AS* (designated as A11AS-PC3) and wild type PC3 cells (Wild); *HOXA11-AS* detection in conditioned medium (CM) of A11AS-PC3 and Wild. (**C**) *HOXA11-AS* expression of SaOS2 cells treated for 48 h with CM of A11AS-PC3 and Wild. (**D**) *CCL2* and *IBSP* expression of SaOS2 cells treated for 48 h with CM of A11AS-PC3 and Wild. qPCR analyses were performed in triplicate (*n* = 3). Expression levels are presented relative to values of *GAPDH*, used as a reference gene, except for the right panel of B. The expression level of *HOXA11-AS* in CM of Wild was set to 1.0 in the right panel of B. Values represent mean ± standard deviation. Statistical significance was determined by unpaired *t*-tests. (**E**) Analysis of the size distribution of isolated EVs from wild type PC3-CM by the NanoSight nanoparticle tracking system (left panel). Comparison of relative abundances of *HOXA11-AS* in PC3 cell, and EV and EV-subtracted supernatant [EV-sup] fractions of PC3-CM by qPCR; equal amounts of RNA were applied (center panel). Relative abundances of *HOXA11-AS* in PC3 cell, and EV and EV-sup fractions are also shown as values per cell and values per mL of CM, respectively (right panel). The expression levels of PC3 were set to 1.0. Data are presented as mean ± standard deviation of triplicate data (*n* = 1).

Extracellular vesicles (EVs) including exosomes, which are small cell-derived vesicles (40–150 nm in diameter), are mediators of the communication between cancer cells and the tumor microenvironment [23]. In a previous work, cellular and exosomal lncRNAs expressed in four prostate cancer cell lines (VCaP, PC3, LNCaP, and DU145) and a normal prostate epithelium cell line (PNT2) were characterized by lncRNA array analysis [24]. We utilized a lncRNA array dataset (GSE81034) to analyze the expression of well-known, cancer metastasis-associated lncRNAs (i.e., *MALAT-1*, *NEAT-1*, *HOTAIR*, and *HOXA11-AS*) in the five cell lines by comparing exosomal RNA versus total RNA (Figure 5A). Notably, *HOXA11-AS* was among the detected lncRNAs in exosomal RNA purified from prostate cancer cell lines (Figure 5A). We hypothesized that *HOXA11-AS* may be transferred from prostate cancer cells to osteoblasts, possibly via EVs, to promote *CCL2* expression in the recipient cells.

To investigate the transfer of PC3-derived *HOXA11-AS* to human osteoblastic osteosarcoma cell SaOS2 cells, we constructed stable clones overexpressing *HOXA11-AS* (designated as A11AS-PC3; Figure 5B). *HOXA11-AS* was significantly highly enriched in conditioned medium (CM) from A11AS-PC3 cells cultured for 48 h compared with control cells (wild type PC3 cells), as detected by qPCR analysis (Figure 5B). After 48 h of culture with A11AS-PC3-CM, *HOXA11-AS* was significantly upregulated in SaOS2 cells (Figure 5C). Moreover, culturing SaOS2 cells with A11AS-PC3-CM for 48 h led to significantly greater expression levels of *CCL2* and *IBSP* (Figure 5D). These results indicate that PC3-derived *HOXA11-AS* is transferred to SaOS2 cells, suggesting that cancer *HOXA11-AS* modulates the expression of osteoblastic cytokine *CCL2* and bone matrix protein *IBSP* to attract cancer cells. 

To assess whether PC3-derived *HOXA11-AS* is present in EVs in PC3-CM, EVs were isolated from wild type PC3-CM in accordance with the differential ultracentrifugation approach described by Thery et al. [12]. PC3 EVs exhibited sizes of 147.8 ± 45.3 nm (mean ± standard deviation) (Figure 5E, left panel). *HOXA11-AS* was present in PC3-derived EVs (Figure 5E, center panel). However, *HOXA11-AS* was enriched in the EV-subtracted supernatant (designated as EV-sup; Figure 5E, right panel), which suggests that non-vesicular forms are equipped to transfer *HOXA11-AS* to the recipient cells.

## 4. Discussion

Cancer transcriptome analysis has identified thousands of lncRNAs that are associated with different types of cancers [25]. Recently, there has been increasing evidence that lncRNAs play fundamental roles in regulation of the immune system; immune-related lncRNAs (including *HOXA11-AS*) involved in cancer have been systematically identified [26,27,28]. Several studies have shown that *HOXA11-AS* exhibited oncogenic roles through different mechanisms in various types of cancers (e.g., breast cancer, lung cancer, liver cancer, gastric carcinoma, and osteosarcoma) [8,9,10,11,29,30,31,32,33,34]. This work is the first to demonstrate that *HOXA11-AS* is highly expressed in prostate cancer cell lines derived from bone metastases (PC3 and VCaP cells), and that it contributes to prostate cancer cell proliferation and invasion. We identified HOXB13 as an upstream regulator of *HOXA11-AS*, and found that *HOXA11-AS* regulated the CCL2/CCR2 signaling associated with prostate cancer bone metastasis. Furthermore, we demonstrated that the HOXB13/*HOXA11-AS* axis regulated *IBSP* promoter and integrin subunits specific to prostate cancer bone metastasis. Finally, we showed that *HOXA11-AS* secreted from PC3 cells could accelerate *CCL2* and *IBSP* expression in recipient SaOS2 osteoblastic cells.

In this study, we identified HOXB13 as an upstream transcriptional regulator of *HOXA11-AS* in human bone metastatic prostate PC3 cells. Deregulation of *HOXB13* expression has been implicated in a variety of other human cancers, functioning as a tumor-promoting factor in some tumor types and a tumor-repressing factor in other tumor types [35,36,37,38,39,40,41,42]. Within the context of HOXB13-associated binding partners, HOXB13 may provide specificity for DNA binding and subsequent tumor progression or repression. In prostate cancer, HOXB13, which is highly expressed in adulthood in the normal prostate [43], functions as a coregulator of AR, together with FOXA1, to modulate the epigenetic status required for prostate tumorigenesis [15,16]. In the present work, we elucidated a novel mechanism for the promotion of prostate cancer bone metastasis, whereby HOXB13 transcriptionally activates and cooperates with *HOXA11-AS*, another putative binding partner of HOXB13. HOXB13 was shown to positively regulate both *HOXA11-AS* and *HOXA11* through a bidirectional promoter. As *HOXA11-AS* repressed *HOXA11*, while *HOXA11* enhanced *HOXA11-AS*, further analyses are necessary to unveil the relation among *HOXA11-AS*, *HOXA11*, and *HOXB13*.

Although we did not experimentally confirm direct regulation of *CDH1* and *MMP3* by *HOXA11-AS*, we found several HOXB13 binding sites in the genomic or intergenic region of these genes though ChIP Atlas database using Integrative Genomics Viewer (IGV) software (Figure S6). Relatively strong HOXB13 binding site (MACS2 score: 739) was found 6 kb upstream the transcription initiation site of *MMP3*. Another HOXB13 binding site (MACS2 score: 607) was found at the 3′ end of *MMP3* gene. In the intronic region of *CDH1* gene, several HOXB13 binding sites (MACS2 score up to 410) were found. It is possible that HOXB13 and *HOXA11-AS,* as a possible HOXB13-binding RNA, regulate the expression of *MMP3* and *CDH1* as well as *IBSP* (Figure S6).

Our findings indicate that the HOXB13/*HOXA11-AS* axis may trigger cytokine and integrin signaling through the modulation of CCL2/CCR2. In small cell lung cancer, CXCL12 induces integrin activation, which results in enhanced adhesion of cancer cells to the extracellular matrix, thereby conferring resistance to chemotherapeutic drugs [44]. Integrins are cell adhesion receptors of the extracellular matrix activated by chemokines that regulate the local microenvironment. They are glycoproteins that form 24 distinct heterodimers from a combination of alpha (α) and beta (β) subunits. At least 18 α (ITGA) and 8 β (ITGB) integrin subunits exist [45,46] Altered integrin expression patterns have been linked to many types of cancer [47,48,49]. Sottnik et al. [50] showed that ITGA2:ITGB1 (α2β1) integrin was elevated in prostate cancer skeletal metastases, compared with either prostate cancer primary lesions or soft tissue metastases; this finding suggests that ITGA2:ITGB1 integrin contributes to selective metastasis to the bone. De et al. [51] reported that ITGAV:ITGB3 (αvβ3) and ITGAV:ITGB5 (αvβ5) integrins were significantly upregulated in bone metastases. Our work indicates that the HOXB13/*HOXA11-AS* axis enhances the expression levels of integrin subunits ITGAV and ITGB1. The results are substantially in agreement with the findings of the previous studies. *HOXA11-AS*-regulated CCL2/CCR2 may modulate the expression of HOXB13/*HOXA11-AS*-regulated integrins in prostate cancer; however, this interaction requires further investigation in future studies.

In this study, the expression of *IBSP*, a gene highly expressed in castration-resistant prostate cancer clinical specimens specifically metastasizing to bone, was found to be positively regulated by HOXB13/*HOXA11-AS*. IBSP is a non-collagenous protein and a member of the small integrin-binding ligand, n-linked glycoprotein family [52]. *IBSP* was found to be expressed by all osteotropic cancers examined, including prostate, lung, thyroid, neuroblastoma, and multiple myeloma; thus, this bone matrix protein might be implicated in the preferential seed and growth of metastatic cells in bone [53,54,55]. Previous reports have shown that the *IBSP* promoter contains a fibroblast growth factor 2 response element (−96 to −89) and a homeodomain protein-binding site (*HOX*; −200 to −191) [56,57], but the involvement of HOXB13 in *IBSP* promoter regulation is a novel finding, denoting the importance of the HOXB13/*HOXA11-AS* axis in prostate cancer bone metastasis.

Many studies have previously investigated cancer–osteoblast interactions in the context of bone metastasis [58,59]. Kimura et al. [60] reported that CM from PC3 cells stimulated calcification in the Tak-10 human osteoblastic cell line. Their findings suggest that some factors generated in prostate cancer cells were secreted and transferred into recipient osteoblasts to modify their function. We demonstrated that *HOXA11-AS* secreted from prostate cancer PC3 cells was able to modify *CCL2* and *IBSP* expression levels in SaOS2 osteoblastic cells in a paracrine manner. Several lncRNAs are associated with extracellular vesicles [61]. Some cancer-related lncRNAs (e.g., *MALAT1, HOTAIR, GAS5*, and *lincRNA-p21*) were found to be enriched in exosomes [62], suggesting that lncRNAs disseminate cell signals and alter the local cellular microenvironment via EVs. In this study, we hypothesized that PC3 cell-derived EVs could serve as a primary mechanism for transfer of *HOXA11-AS* to SaOS2 cells. Although EVs might partially contribute to the prostate cancer–osteoblast interaction (Figure 5E), PC3-derived *HOXA11-AS* was presumably transferred to SaOS2 cells in an extracellular vesicle-independent manner. Recently, a novel population of non-membranous nanoparticles, termed exomeres (~35 nm in diameter), was discovered by asymmetric flow field-flow fractionation [63]. Exomeres exhibit a distinct cargo protein profile compared with EVs; exomeres show enriched levels of metabolic enzymes (e.g., MAT1A1 and ST6Gal-I), Argonaute proteins, and amyloid precursor protein [63,64]. Zhang et al. [64] showed that exomere cargos (i.e., ST6Gal-I and EGFR ligand amphiregulin) could be transferred to cancer cells, resulting in modified biological activities of recipient cells. Future studies are needed to identify the main transport systems that permit the transfer of PC3-derived *HOXA11-AS* to recipient SaOS2 cells.

**Figure 6:**
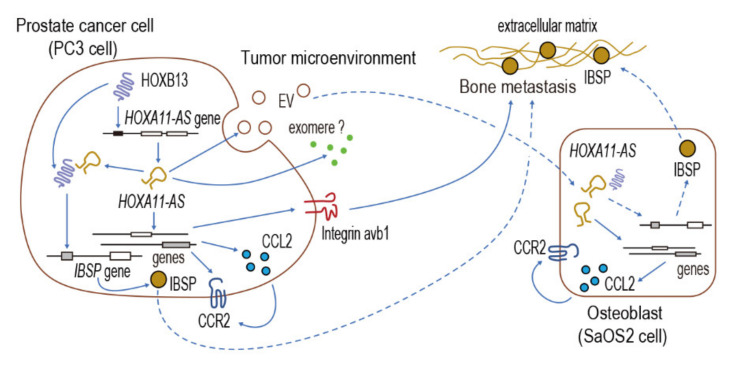
Working model of HOXB13 and *HOXA11-AS* for promotion of prostate cancer bone metastasis. HOXB13/*HOXA11-AS* axis regulates integrin and CCL2/CCR2 cytokine signaling in prostate cancer cells, while paracrine action of *HOXA11-AS*-secreted from prostate cancer PC3 cells is able to modulate CCL2/CCR2 cytokine signaling in osteoblasts within the bone marrow milieu.

In conclusion, our study developed a unique model that may explain the interrelationship among lncRNAs, transcription factors, chemokines, and integrins, resulting in the high osteotropism of prostate cancer metastasis (Figure 6). Our results suggest that the lncRNA, *HOXA11-AS*, together with its transcription factor, HOXB13, regulate the osteotropism of prostate cancer cells through specific downstream cytokine and integrin signals. Although further studies are needed to demonstrate the orchestrated function of *HOXA11-AS* and HOXB13, additional research regarding the signaling pathways that are intrinsic to the invasive and migratory phenotype of prostate cancer holds promise for the development of novel cancer therapeutics with greater efficacy.

## Data Availability

Data available in a publicly accessible repository. The data presented in this study are openly available in the Gene Expression Omnibus database at https://www.ncbi.nlm.nih.gov/geo, reference number GSE147710.

## Supplementary Materials

The following are available online at https://www.mdpi.com/2073-4425/12/2/182/s1, Figure S1: AR is not involved in the regulation of HOXA11-AS, Figure S2: FOXA1 is not involved in the regulation of HOXA11-AS, Figure S3: HOXB13 knockdown downregulates HOXA11-AS. Figure S4: HOXB13 knockdown downregulates CCR2, Figure S5: HOXB13 and HOXA11-AS knockdown modulates protein levels of integrin subunits, Figure S6: HOXB13 binding sites on MMP3 and CDH1 gene locus in prostate cancer cells, Table S1: PCR primers used for plasmid construction, Table S2. siRNAs used for gene silencing, Table S3. PCR primers used for qRT-PCR analysis, Table S4. Membrane associated genes commonly upregulated by HOXA11-AS and HOXB13 knockdown, Table S5. Cytokines involved in the progression of prostate cancer bone metastasis.
