# Supplementary material for: Long Noncoding RNA HOXA11-AS and Transcription Factor HOXB13 Modulate the Expression of Bone Metastasis-Related Genes in Prostate Cancer
Source: Genes (Basel). 2021 Jan 27;12(2):182. doi: 10.3390/genes12020182 (PMC7912412; doi:10.3390/genes12020182)
Supplement: Supplementary file 1 [file genes-12-00182-s001.zip › Genes Suppl Figs and Tables ver210126/Genes Suppl Tables S1-S5 ver210126 with security.docx]

**Supplementary Tables S1-S5**

**Table S1. PCR primers used for plasmid construction**

| **HOXA11-AS vector** | **Forward Primer** | **Reverse Primer** |
| --- | --- | --- |
| HOXA11-AS vector | CGC*AAGCTT*GCCACCTCAGGGGAAGCAACAGAT | CGC*GGTACC*GGCGCCTGGACCCACTTTATTCCG |
|  |  |  |
| **Luciferase vectors** | **Forward Primer** | **Reverse Primer** |
| IBSP-Promoter | CGC*GGTACC*CCTCTTGGCTCTAGAATCACG | CGC*GAGCTC*AAATCTTACCCTCTGGCAGTCCTG |
| HOXA11-AS Promoter (+) | CGC*GGTACC*TAGAGCTAAGCGGGCTACTTTATC | CGC*AAGCTT*CGGAGGCAGAGGCGCTTCCCAGAG |
| HOXA11-AS Promoter (-) | CGC*GGTACC*GGCAGAAAAGAAGGGAGGCTGGAG | CGC*AAGCTT*CGGAGGCAGAGGCGCTTCCCAGAG |

Restriction enzyme sites are shown in *italics.*

**Table S2. siRNAs used for gene silencing**

| **siRNA** | **Target sequence** |
| --- | --- |
| siHOXA11-AS (1) | GAGTGTATTTCCTTTTATCGTCA |
| siHOXA11-AS (2) | CGGCTAACAAGGAGATTTGGTCA |
| siHOXA11-AS (3) | TTCTTGTTTTTCCAAACTTCAAG |
| siHOXA11 (1) | AAGATAAATATTATATTATATAT |
| siHOXA11 (2) | TTCAAAAAACTTGTAAATAAAGG |
| siHOXB13 (1) | TTCATGAATTGAGCTAATTATGA |
| siHOXB13 (2) | GAGCTAATTATGAATAAATTTGG |
| siIBSP (1) | AAGAATTTCATTGACATTAATGA |
| siIBSP (2) | ACGAGTTTTATCACAAATAAAAA |
| siAR (1) | AACAAAACAAACAAAAACAAAAA |
| siAR (2) | AGGATTTTGTTTTTTTCTTTTAA |

**Table S3. PCR primers used for qRT-PCR analysis**

| **Gene** | **Forward Primer** | **Reverse Primer** |
| --- | --- | --- |
| *HOXA11* | AAGCGCTGCCCCTATACCAA | CAGTGAGGTTGAGCATGCGG |
| *HOXA11-AS* (1) | CCACCCATCTGCCTGGTCTT | AAGTCTCCTCGGATGTCAGCG |
| *HOXA11-AS* (2) | GCGCGTAAGGCTGTGACATT | GTTTTCTGGAGATGGCCCGC |
| *HOXB13* | GCCAGGGAGAACAGAACCCA | TGCCCCTTGCTGTACGGAAT |
| *CDH1* | AGCCCGAATTCACCCAGGAG | ACATCATCGTCCGCGTCTGT |
| *MMP3* | TTGGATTGGAGGTGACGGGG | TTCCTCCACTTCGGGATGCC |
| *IBSP* (1) | CAGGACTGCCAGAGGAAGCA | GAAAGCACAGGCCATTCCCA |
| *IBSP* (2) | TTACTACCACCACCAGTGAAGC | GATGCAAAGCCAGAATGGAT |
| *IBSP* for ChIP assay | GGGCAGTAGTGACTCATCCGA | TCTTGTGCTGTCTGCTTTATTACGA |
| *ITGA2B* | TGGTGGTGGCAGCAGAAGAA | AGGGCCATTGTTGTGGAGCT |
| *ITGA2* | ACCGAGGTGACCAGATTGGC | GGTGCTGACCCAAAATGCCC |
| *ITGA5* | TCCTGTTTGGCCTCCTGCTC | GCCATATGGGAGGGAGCGTT |
| *ITGA8* | AGTTGTGGCCTGTGCTCCTT | TGGCCTTCCGGATCAGCATT |
| *ITGAV* | TTTACTGGCGAGCAGATGGC | GTTTGCCATCAGAGCCACGA |
| *ITGB1* | CCAAATTGTGGGTGGTGCAC | GGCTTGAGCTTCTCTGCTGT |
| *ITGB3* | TGCCTGGTGCTCTGATGAGG | GCTGTCTCCAGAGCCCTTGT |
| *ITGB5* | ATGCAGTACTCCAGGCAGCC | CATCCAATGCGATGTGGGGC |
| *ITGB6* | GACACAAAGGGCTGCTCTGC | TGCAGTACTCGCCAGTCCAG |
| *ITGB8* | AGTGGTGCCCAATGACGGAA | GCAATGGTGCCTGGCAAGAG |
| *CCL2* | AAGTCTCTGCCGCCCTTCTG | GCGAGCCTCTGCACTGAGAT |
| *CCR2* | GCAAATTGGGGCCCAACTCC | AGATGGCCAGGTTGAGCAGG |
| *GAPDH* | AAGGCTGTGGGCAAGGTCAT | CAGGTCCACCACTGACACGT |

**Table S4. Membrane associated genes commonly upregulated by *HOXA11-AS* and *HOXB13* knockdown**

| **Gene Symbol** | **Gene Name** |
| --- | --- |
| *ABCB10* | *ATP binding cassette subfamily B member 10* |
| *ATP6AP2* | *ATPase H+ transporting accessory protein 2* |
| *ATP2B4* | *ATPase plasma membrane Ca2+ transporting 4* |
| *AXL* | *AXL receptor tyrosine kinase* |
| *ACAP2* | *ArfGAP with coiled-coil, ankyrin repeat and PH domains 2* |
| *BNIP3* | *BCL2 interacting protein 3* |
| *CD46* | *CD46 molecule* |
| *DCAF17* | *DDB1 and CUL4 associated factor 17* |
| *EFHD2* | *EF-hand domain family member D2* |
| *EFR3A* | *EFR3 homolog A* |
| *ERRFI1* | *ERBB receptor feedback inhibitor 1* |
| *GNG5* | *G protein subunit gamma 5* |
| *GPR176* | *G protein-coupled receptor 176* |
| *GLIPR1* | *GLI pathogenesis related 1* |
| *GRAMD1B* | *GRAM domain containing 1B* |
| *LEMD2* | *LEM domain containing 2* |
| *PNLDC1* | *PARN like, ribonuclease domain containing 1* |
| *RAB1A* | *RAB1A, member RAS oncogene family* |
| *TBC1D5* | *TBC1 domain family member 5* |
| *B3GNT5* | *UDP-GlcNAc:betaGal beta-1,3-N-acetylglucosaminyltransferase 5* |
| *VPS33A* | *VPS33A, CORVET/HOPS core subunit* |
| *AP1S3* | *adaptor related protein complex 1 sigma 3 subunit* |
| *AP2A1* | *adaptor related protein complex 2 alpha 1 subunit* |
| *ANKRD13C* | *ankyrin repeat domain 13C* |
| *AREL1* | *apoptosis resistant E3 ubiquitin protein ligase 1* |
| *ATG2B* | *autophagy related 2B* |
| *BLCAP* | *bladder cancer associated protein* |
| *C4orf32* | *chromosome 4 open reading frame 32* |
| *DSEL* | *dermatan sulfate epimerase-like* |
| *EMP3* | *epithelial membrane protein 3* |
| *EPB41L3* | *erythrocyte membrane protein band 4.1 like 3* |
| *GNPNAT1* | *glucosamine-phosphate N-acetyltransferase 1* |
| *INPP5A* | *inositol polyphosphate-5-phosphatase A* |
| *ITGAM* | *integrin subunit alpha M* |
| *LMAN1* | *lectin, mannose binding 1* |
| *LCLAT1* | *lysocardiolipin acyltransferase 1* |
| *MGLL* | *monoglyceride lipase* |
| *MUC13* | *mucin 13, cell surface associated* |
| *NRSN2* | *neurensin 2* |
| *NEGR1* | *neuronal growth regulator 1* |
| *NIPA1* | *non imprinted in Prader-Willi/Angelman syndrome 1* |
| *OCLM* | *oculomedin* |
| *PIK3C2B* | *phosphatidylinositol-4-phosphate 3-kinase catalytic subunit* |
| *PLEK2* | *pleckstrin 2* |
| *PODXL* | *podocalyxin like* |
| *KCNMA1* | *potassium calcium-activated channel subfamily M alpha 1* |
| *PTK2* | *protein tyrosine kinase 2* |
| *PTPN1* | *protein tyrosine phosphatase, non-receptor type 1* |
| *P2RY2* | *purinergic receptor P2Y2* |
| *RMDN3* | *regulator of microtubule dynamics 3* |
| *RRAS2* | *related RAS viral* |
| *RECK* | *reversion inducing cysteine rich protein with kazal motifs* |
| *RNFT1* | *ring finger protein, transmembrane 1* |
| *SEMA4B* | *semaphorin 4B* |
| *SEMA7A* | *semaphorin 7A* |
| *SGK1* | *serum/glucocorticoid regulated kinase 1* |
| *SCNN1G* | *sodium channel epithelial 1 gamma subunit* |
| *SCN9A* | *sodium voltage-gated channel alpha subunit 9* |
| *SLC1A3* | *solute carrier family 1 member 3* |
| *SLC37A3* | *solute carrier family 37 member 3* |
| *SLC7A6* | *solute carrier family 7 member 6* |
| *SGPL1* | *sphingosine-1-phosphate lyase 1* |
| *SYNPO* | *synaptopodin* |
| *TTC7B* | *tetratricopeptide repeat domain 7B* |
| *TRHDE* | *thyrotropin releasing hormone degrading enzyme* |
| *TFRC* | *transferrin receptor* |
| *TMBIM6* | *transmembrane BAX inhibitor motif containing 6* |
| *TMEM158* | *transmembrane protein 158* |
| *TMEM225* | *transmembrane protein 225* |
| *TMEM243* | *transmembrane protein 243* |
| *TULP3* | *tubby like protein 3* |
| *UBE2J1* | *ubiquitin conjugating enzyme E2 J1* |
| *VAMP7* | *vesicle associated membrane protein 7* |

**Table S5. Cytokines involved in the progression of prostate cancer bone metastasis**

| **Chemokine ligand/receptor** | **Function** | **Chemokine antagonist** | **References** |
| --- | --- | --- | --- |
| CCL2 / CCR2 | Bone resorption, tumor growth in bone | CNTO-888 / C1142 (anti-CCL2 antibody) | (Lu et al., 2009), (Lu et al., 2007) |
| CCL3, 4, 5 3L1 / CCR5 | Osteolysis | Maraviroc | (Sicoli et al., 2014) |
| CXCL1, 2 / CXCR2 | Paracrine action on endothelial cells and osteoblasts, osteolysis | Anti-CXCL1 antibody | (Hardaway, Herroon, Rajagurubandara, & Podgorski, 2015), (Lee et al., 2015) |
| CXCL8 (IL8) / CXCR1, 2 | Osteolysis, tumor growth in bone | SCH527123 (CXCR1/2 antagonist) | (Lu et al., 2007), (Lee et al., 2011), (Lu et al., 2019) |
| CXCL12 / CXCR4 | Trafficking and migration to bone, tumor growth in bone | Anti-CXCR4 antibody | (Taichman et al., 2002), (Sun et al., 2005) |
| CXCL12 / CXCR4  CXCL16 / CXCR6 | Trafficking and migration to bone, tumor growth in bone  cytoskeleton rearrangement and αvβ3 integrin clustering to promote metastasis | Plerixafor / AMD3100 | (Gravina et al., 2015) |
|  |  | CTCE-9908 | (Domanska et al., 2012), (Conley-LaComb et al., 2016),(Jung et al., 2013) |
|  |  | Anti-CXCL16 antibody | (Singh et al., 2016) |

Conley-LaComb, M. K., Semaan, L., Singareddy, R., Li, Y., Heath, E. I., Kim, S., . . . Chinni, S. R. (2016). Pharmacological targeting of CXCL12/CXCR4 signaling in prostate cancer bone metastasis. *Mol Cancer, 15*(1), 68. doi:10.1186/s12943-016-0552-0

Domanska, U. M., Timmer-Bosscha, H., Nagengast, W. B., Oude Munnink, T. H., Kruizinga, R. C., Ananias, H. J., . . . Walenkamp, A. M. (2012). CXCR4 inhibition with AMD3100 sensitizes prostate cancer to docetaxel chemotherapy. *Neoplasia, 14*(8), 709-718. doi:10.1593/neo.12324

Gravina, G. L., Mancini, A., Muzi, P., Ventura, L., Biordi, L., Ricevuto, E., . . . Festuccia, C. (2015). CXCR4 pharmacogical inhibition reduces bone and soft tissue metastatic burden by affecting tumor growth and tumorigenic potential in prostate cancer preclinical models. *Prostate, 75*(12), 1227-1246. doi:10.1002/pros.23007

Hardaway, A. L., Herroon, M. K., Rajagurubandara, E., & Podgorski, I. (2015). Marrow adipocyte-derived CXCL1 and CXCL2 contribute to osteolysis in metastatic prostate cancer. *Clin Exp Metastasis, 32*(4), 353-368. doi:10.1007/s10585-015-9714-5

Jung, Y., Kim, J. K., Shiozawa, Y., Wang, J., Mishra, A., Joseph, J., . . . Taichman, R. S. (2013). Recruitment of mesenchymal stem cells into prostate tumours promotes metastasis. *Nat Commun, 4*, 1795. doi:10.1038/ncomms2766

Lee, Y. C., Cheng, C. J., Bilen, M. A., Lu, J. F., Satcher, R. L., Yu-Lee, L. Y., . . . Lin, S. H. (2011). BMP4 promotes prostate tumor growth in bone through osteogenesis. *Cancer Res, 71*(15), 5194-5203. doi:10.1158/0008-5472.CAN-10-4374

Lee, Y. C., Gajdosik, M. S., Josic, D., Clifton, J. G., Logothetis, C., Yu-Lee, L. Y., . . . Lin, S. H. (2015). Secretome analysis of an osteogenic prostate tumor identifies complex signaling networks mediating cross-talk of cancer and stromal cells within the tumor microenvironment. *Mol Cell Proteomics, 14*(3), 471-483. doi:10.1074/mcp.M114.039909

Lu, Y., Cai, Z., Xiao, G., Keller, E. T., Mizokami, A., Yao, Z., . . . Zhang, J. (2007). Monocyte chemotactic protein-1 mediates prostate cancer-induced bone resorption. *Cancer Res, 67*(8), 3646-3653. doi:10.1158/0008-5472.CAN-06-1210

Lu, Y., Chen, Q., Corey, E., Xie, W., Fan, J., Mizokami, A., & Zhang, J. (2009). Activation of MCP-1/CCR2 axis promotes prostate cancer growth in bone. *Clin Exp Metastasis, 26*(2), 161-169. doi:10.1007/s10585-008-9226-7

Lu, Y., Dong, B., Xu, F., Xu, Y., Pan, J., Song, J., . . . Xue, W. (2019). CXCL1-LCN2 paracrine axis promotes progression of prostate cancer via the Src activation and epithelial-mesenchymal transition. *Cell Commun Signal, 17*(1), 118. doi:10.1186/s12964-019-0434-3

Sicoli, D., Jiao, X., Ju, X., Velasco-Velazquez, M., Ertel, A., Addya, S., . . . Pestell, R. G. (2014). CCR5 receptor antagonists block metastasis to bone of v-Src oncogene-transformed metastatic prostate cancer cell lines. *Cancer Res, 74*(23), 7103-7114. doi:10.1158/0008-5472.CAN-14-0612

Singh, R., Kapur, N., Mir, H., Singh, N., Lillard, J. W., Jr., & Singh, S. (2016). CXCR6-CXCL16 axis promotes prostate cancer by mediating cytoskeleton rearrangement via Ezrin activation and alphavbeta3 integrin clustering. *Oncotarget, 7*(6), 7343-7353. doi:10.18632/oncotarget.6944

Sun, Y. X., Schneider, A., Jung, Y., Wang, J., Dai, J., Wang, J., . . . Taichman, R. S. (2005). Skeletal localization and neutralization of the SDF-1(CXCL12)/CXCR4 axis blocks prostate cancer metastasis and growth in osseous sites in vivo. *J Bone Miner Res, 20*(2), 318-329. doi:10.1359/JBMR.041109

Taichman, R. S., Cooper, C., Keller, E. T., Pienta, K. J., Taichman, N. S., & McCauley, L. K. (2002). Use of the stromal cell-derived factor-1/CXCR4 pathway in prostate cancer metastasis to bone. *Cancer Res, 62*(6), 1832-1837. Retrieved from <https://www.ncbi.nlm.nih.gov/pubmed/11912162>
