# Supplementary figures and images for: Long Noncoding RNA HOXA11-AS and Transcription Factor HOXB13 Modulate the Expression of Bone Metastasis-Related Genes in Prostate Cancer
Source: Genes (Basel). 2021 Jan 27;12(2):182. doi: 10.3390/genes12020182 (PMC7912412; doi:10.3390/genes12020182)

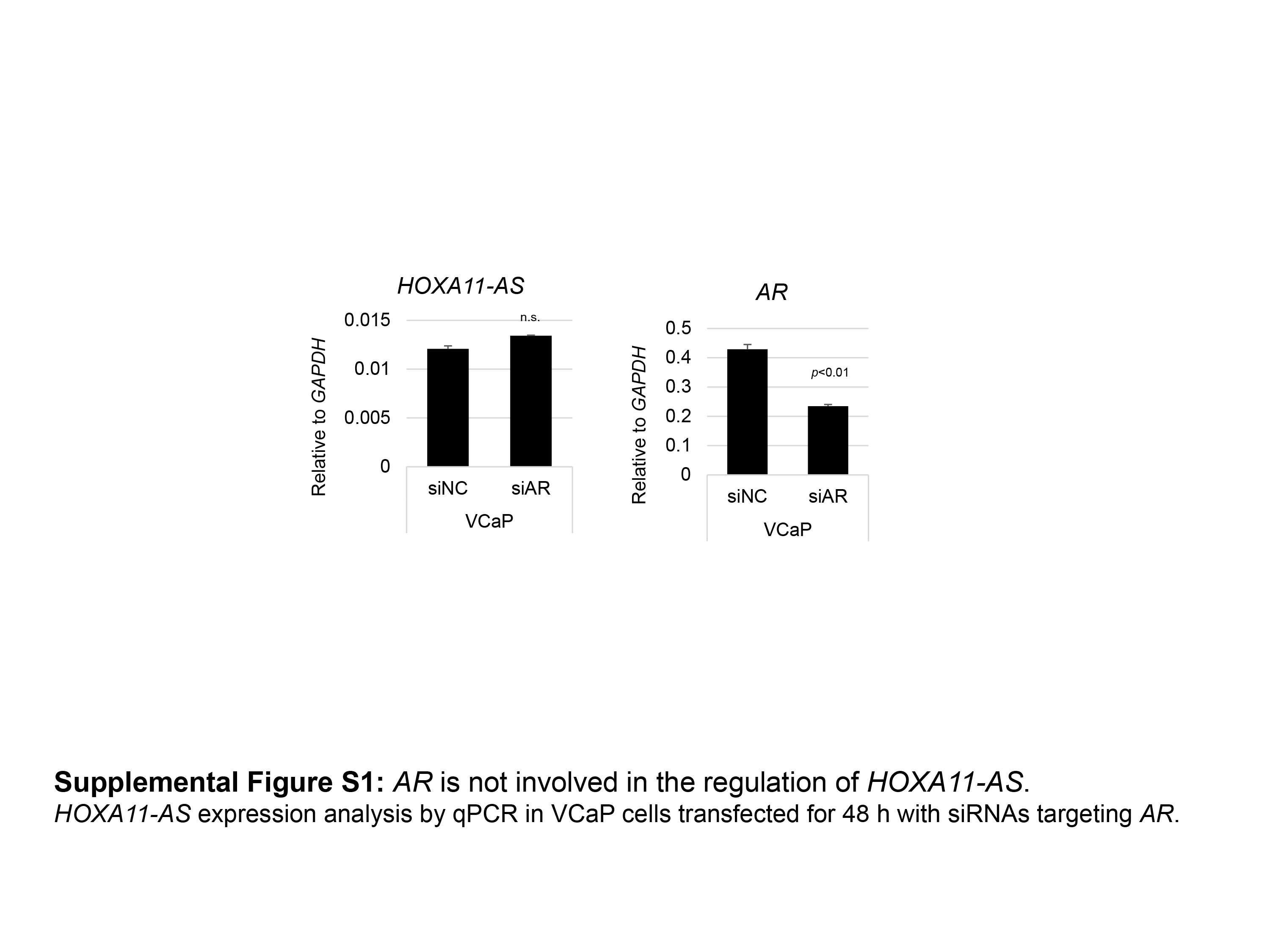

Supplement: Supplementary file 1 [file genes-12-00182-s001.zip › Genes Suppl Figs and Tables ver210126/Gens Suppl Fig S1 ver210126 A.tif]

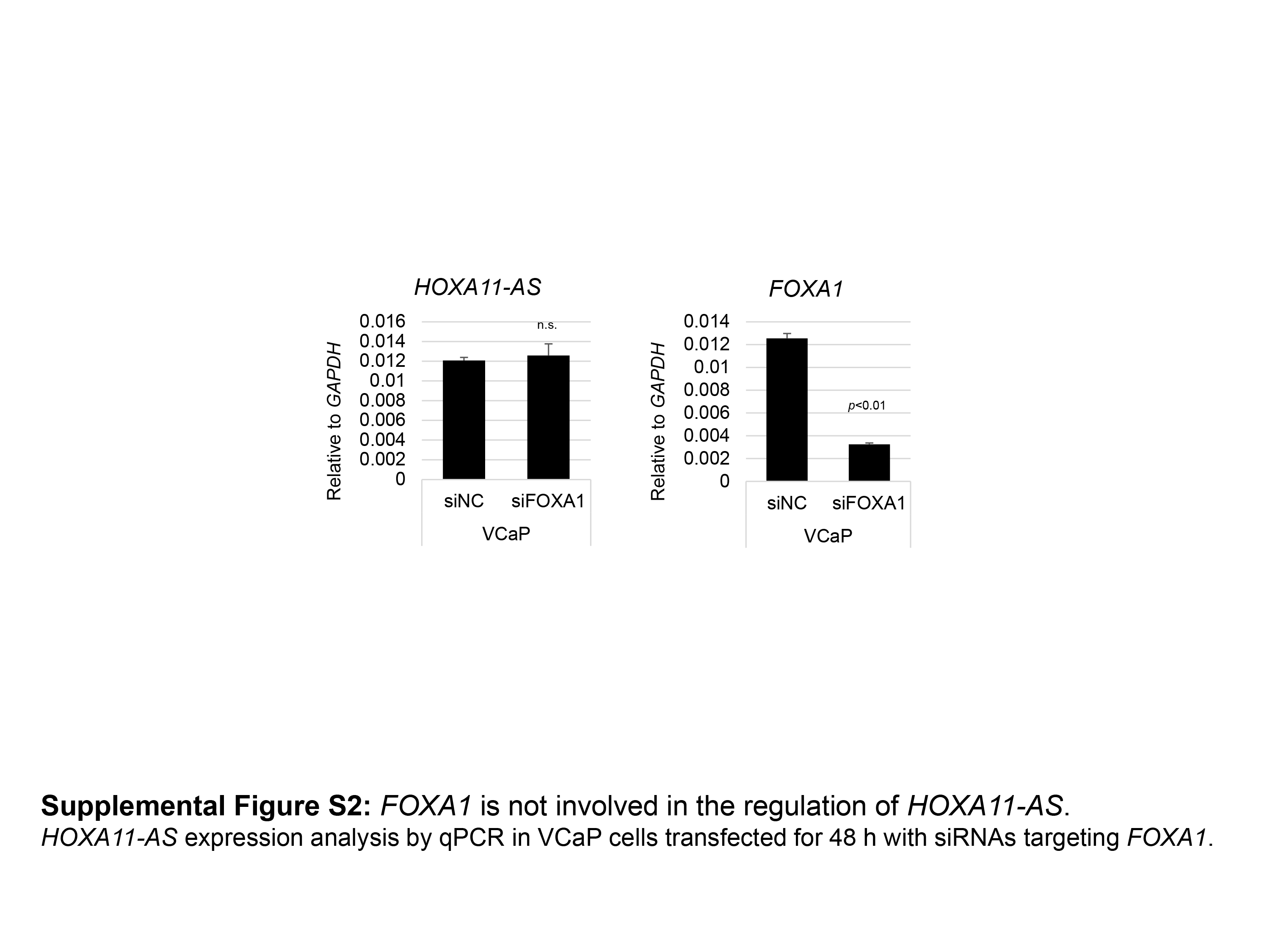

Supplement: Supplementary file 1 [file genes-12-00182-s001.zip › Genes Suppl Figs and Tables ver210126/Gens Suppl Fig S2 ver210126 A.tif]

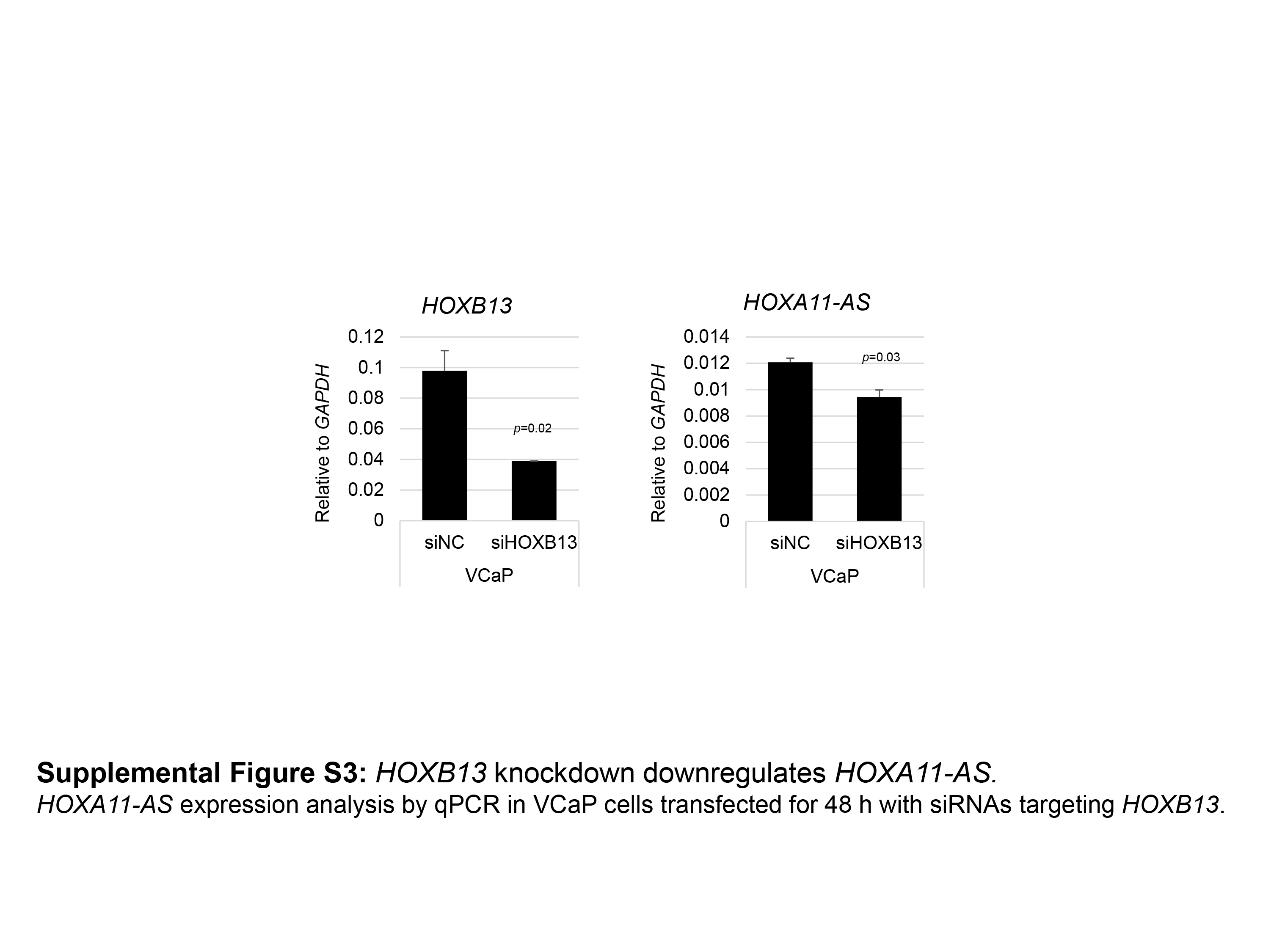

Supplement: Supplementary file 1 [file genes-12-00182-s001.zip › Genes Suppl Figs and Tables ver210126/Gens Suppl Fig S3 ver210126 A.tif]

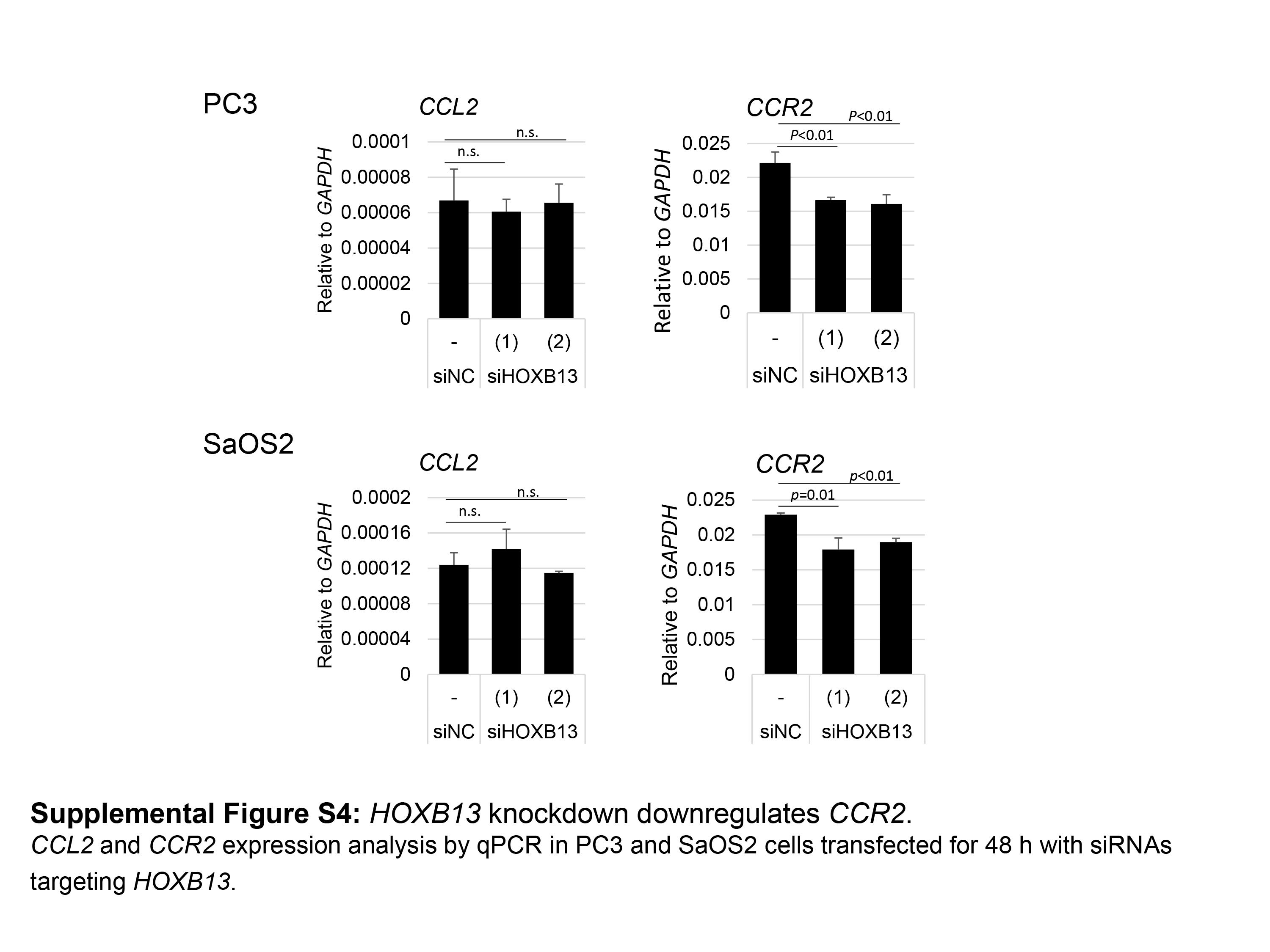

Supplement: Supplementary file 1 [file genes-12-00182-s001.zip › Genes Suppl Figs and Tables ver210126/Gens Suppl Fig S4 ver210126 A.tif]

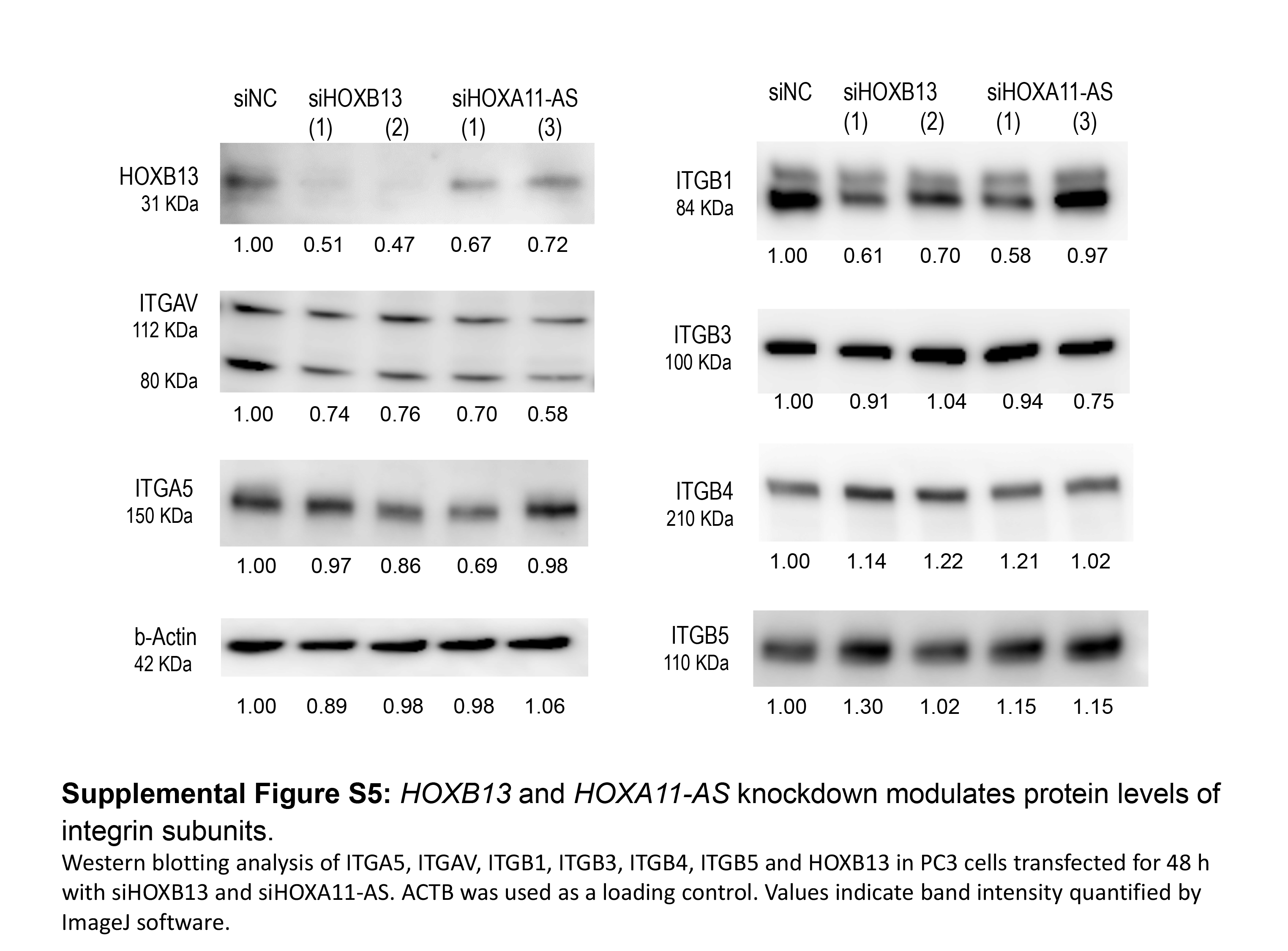

Supplement: Supplementary file 1 [file genes-12-00182-s001.zip › Genes Suppl Figs and Tables ver210126/Gens Suppl Fig S5 ver210126 A.tif]

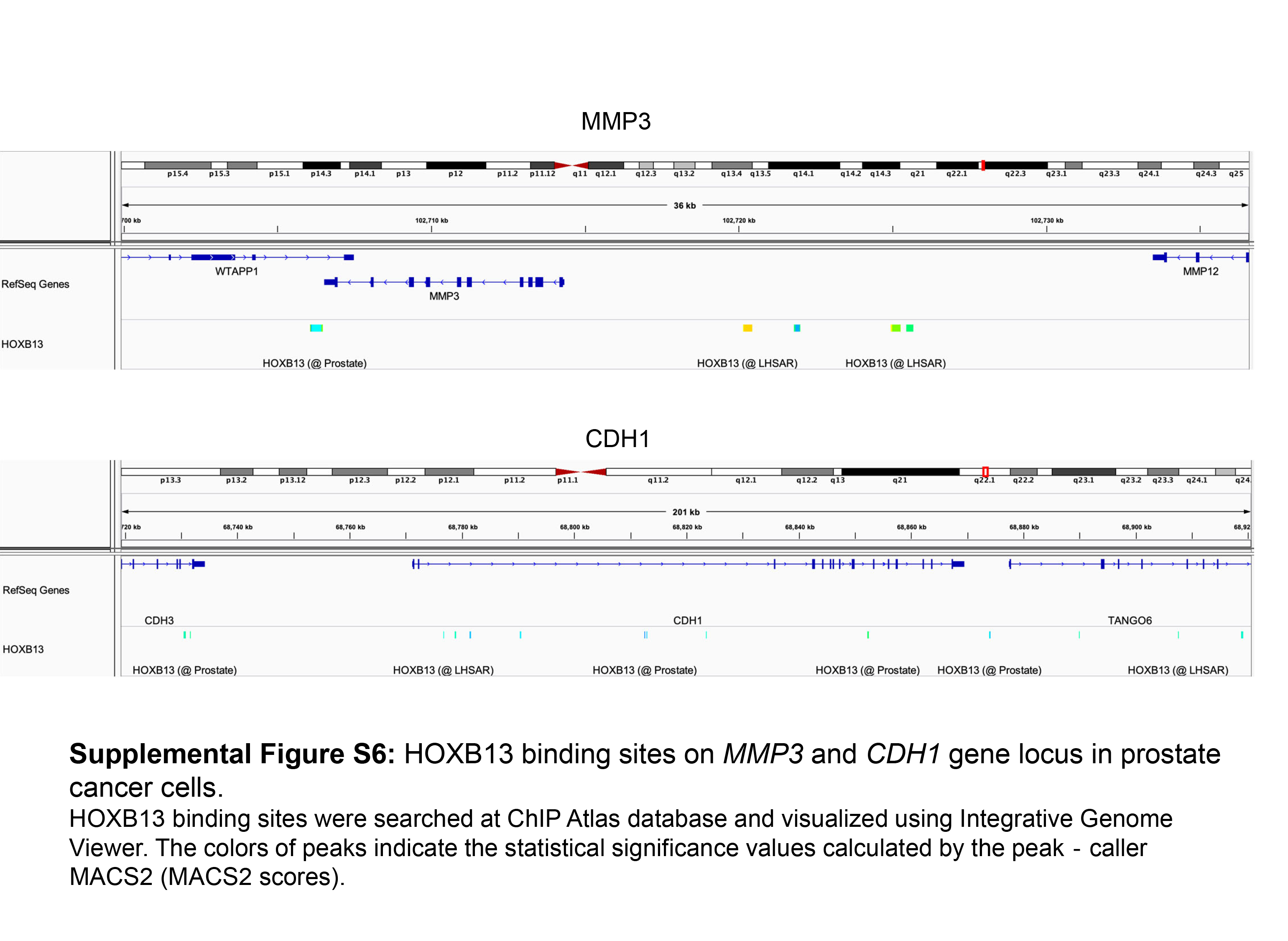

Supplement: Supplementary file 1 [file genes-12-00182-s001.zip › Genes Suppl Figs and Tables ver210126/Gens Suppl Fig S6 ver210126 A.tif]
